# Getting the ecology into interactions between plants and the plant growth-promoting bacterium *Pseudomonas fluorescens*

**DOI:** 10.3389/fpls.2013.00081

**Published:** 2013-04-10

**Authors:** W. H. Gera Hol, T. Martijn Bezemer, Arjen Biere

**Affiliations:** Department of Terrestrial Ecology, Netherlands Institute of EcologyWageningen, Netherlands

**Keywords:** PGPR-bacteria, *Pseudomonas fluorescens*, species interactions, complexity, herbivores, decomposers, bacterivores, mutualists

## Abstract

Plant growth-promoting rhizobacteria (PGPR) are increasingly appreciated for their contributions to primary productivity through promotion of growth and triggering of induced systemic resistance in plants. Here we focus on the beneficial effects of one particular species of PGPR (*Pseudomonas fluorescens*) on plants through induced plant defense. This model organism has provided much understanding of the underlying molecular mechanisms of PGPR-induced plant defense. However, this knowledge can only be appreciated at full value once we know to what extent these mechanisms also occur under more realistic, species-diverse conditions as are occurring in the plant rhizosphere. To provide the necessary ecological context, we review the literature to compare the effect of *P. fluorescens* on induced plant defense when it is present as a single species or in combination with other soil dwelling species. Specifically, we discuss combinations with other plant mutualists (bacterial or fungal), plant pathogens (bacterial or fungal), bacterivores (nematode or protozoa), and decomposers. Synergistic interactions between *P. fluorescens* and other plant mutualists are much more commonly reported than antagonistic interactions. Recent developments have enabled screenings of *P. fluorescens* genomes for defense traits and this could help with selection of strains with likely positive interactions on biocontrol. However, studies that examine the effects of multiple herbivores, pathogens, or herbivores and pathogens together on the effectiveness of PGPR to induce plant defenses are underrepresented and we are not aware of any study that has examined interactions between *P. fluorescens* and bacterivores or decomposers. As co-occurring soil organisms can enhance but also reduce the effectiveness of PGPR, a better understanding of the biotic factors modulating *P. fluorescens*–plant interactions will improve the effectiveness of introducing *P. fluorescens* to enhance plant production and defense.

## INTRODUCTION

Plant growth-promoting rhizobacteria (PGPR) are a diverse group of microorganisms that are increasingly appreciated for their contributions to primary productivity through promotion of growth and triggering of induced systemic resistance (ISR) in plants. By triggering plant defense, PGPR can make an important contribution to biocontrol of pests and pathogens of plants. However, the effectiveness of PGPR-triggered plant defense depends on a variety of genetic and biotic/abiotic environmental factors. PGPR naturally occur within a complex community of soil organisms inhabiting the rhizosphere. Hence, in order to understand the role of PGPR in influencing a plant’s defense against pests and pathogens, it is important to understand how biotic interactions with these rhizosphere organisms will affect the ability of PGPR to enhance plant defense. The aim of this review is to examine how the impact of PGPR on plant defense is modulated by the presence of other organisms in the rhizosphere. Other reviews have focused on particular interactions, e.g., between PGPR and aboveground insects (Pieterse and Dicke, 2007; Pineda et al., 2010) or type of defense, e.g., volatiles (Dicke and Baldwin, 2010). Those reviews have taken a plant centric approach (but see Whipps, 2001). In this paper, we will review how biotic interactions between PGPR and other rhizosphere- or plant-associated organisms affect the ability of PGPR to enhance plant defenses. We use *Pseudomonas fluorescens*, a very common and well-studied PGPR, as a model species. To test the dependence of PGPR–plant interactions on direct and indirect biotic interactions with other rhizosphere biota, we compare studies in which effects of *P. fluorescens* on plant defense are examined for a single *P. fluorescens* isolate with studies in which these effects are examined for a *P. fluorescens* isolate in combination with other isolates and/or species. We will discuss these interactions in increasing order of complexity, starting with single introductions of *P. fluorescens* with introductions of multiple *P. fluorescens* isolates, then with other PGPR, with other plant growth-promoting fungi, bacterivores, and finally with decomposing organisms. The basic interaction in all these studies is formed by a plant, *P. fluorescens* and a herbivore or pathogen. The latter is necessary to judge whether plant defense was changed. In addition, studies without herbivore or pathogens but that measure plant defense genes are included. Before we review these interactions we provide a brief introduction to PGPR and *P. fluorescens* in particular. Moreover, as we argue that the effect of PGPR on induced plant defense cannot be considered in isolation from the effects of other organisms that are also present in the soil such as nematodes, fungi, earthworms, or protozoa on the PGPR or on the plant, we also provide a brief overview of interactions between bacteria and other soil dwelling organisms in the rhizosphere.

## INTERACTIONS BETWEEN BACTERIA AND OTHER SOIL ORGANISMS IN THE RHIZOSPHERE

Live roots and root exudates provide a diverse range of resources to soil organisms. As a result, the zone around plant roots, the rhizosphere, is a highly diverse habitat. It consists of root herbivores, such as nematodes and insect larvae, their natural enemies, and a wide variety of soil microbes, including symbiotic, pathogenic, and saprophytic fungi and protozoa. The vast majority of soil organisms in the rhizosphere are bacteria (including PGPR), with densities as high as 10^9^ cells per gram of soil. The abundance and composition of these soil bacteria depends on abiotic conditions such as soil pH, temperature, and moisture (Bardgett, 2005). However, in the rhizosphere of plants, the density and activity of bacteria is fuelled largely by root-derived carbon. Bacteria compete with each other and other soil microorganisms for these carbon resources.

In the rhizosphere, bacteria can have direct beneficial or harmful effects on the plant. However, there are also important indirect feedback interactions between plant roots, soil bacteria, and other microorganisms (Berendsen et al., 2012). For example, root-released exudates promote bacterial growth (Bais et al., 2001). These bacteria are consumed by protozoa and bacterivorous nematodes, and these consumers generally cause strong top-down control of bacteria. Via bacterial grazing, these bacterivores liberate nutrients, which in turn, stimulate plant growth (Bonkowski, 2004). The quality and quantity of root-derived carbon sources vary temporally, between plant species and between individual plants that belong to the same plant species. This variation can be attributed, at least partly, to interactions between plants and other organisms. Foliar herbivory, but also interactions between roots and soil organisms such as root herbivores or mycorrhizal fungi (Jones et al., 2004; Bais et al., 2006), often causes an increase in the rate of carbon and nitrogen exudation from roots which then leads to enhanced microbial activity in the rhizosphere (Holland et al., 1996; Bardgett and Wardle, 2010). Hence, bacterial growth and activity will depend on the direct and indirect interactions of the plant with other (soil) organisms. Apart from consumption, movement of larger soil fauna also affects the dispersal of soil microorganisms such as bacteria. Thus, plant roots, bacteria, and other microbes interact in complex food webs and in order to understand the interactions between plants and bacteria it is important to consider them in a multitrophic context.

## PGPR AND PLANT DEFENSE

Rhizobacteria with growth-promoting capacity occur in a number of bacterial phyla (Actinobacteria, Bacteroidetes, Cyanobacteria, Firmicutes, Proteobacteria) with as best known members *Pseudomonas* spp. and *Bacillus* spp. (Compant et al., 2005). Studies on model organisms like* Pseudomonas fluorescens* have provided considerable understanding of the underlying molecular mechanisms of PGPR-induced plant defense. The type of defense triggered by microorganisms differs among pathogenic and non-pathogenic microbes (van Loon, 2007; Pieterse et al., 2012). Biotrophic pathogens generally induce systemic acquired resistance (SAR). SAR is dependent on salicylic acid (SA) signaling and results in enhanced expression of pathogenesis-related (PR) proteins (Durrant and Dong, 2004). By contrast, PGPR trigger ISR. ISR is generally independent of the SA signaling pathway and is not associated with major alterations in the expression of defense-related genes, but with priming of defenses (Verhagen et al., 2004). PGPR-primed plants do not have elevated expression of defense genes. Instead, they show more rapid or stronger activation of defenses once they are attacked by pathogens and herbivores, a response that is often dependent on a functional JA pathway (van der Ent et al., 2009). ISR is a systemic response, expressed in both roots and shoots, that can affect a wide range of organisms, including above- and belowground pathogens, herbivores, and their natural enemies (Pineda et al., 2010, 2012), a spectrum that only partly overlaps with that of SAR (van Oosten et al., 2008).

The effect of PGPR on plant growth promotion and on plant defense against attackers depends on many factors, including the plant species or genotype, the pathogen species, and the abiotic conditions, such as nutrient availability. In some cases variation in these factors can even lead to opposite effects of PGPR on plant traits. For instance, *P. fluorescens* addition stimulated nitrogen mineralization in one crop species and decreased it in another (Brimecombe et al., 1999). Similar variation in the effects of PGPR among plant species has been observed for their effects on plant defense against pathogens and herbivores. For instance, Tétard-Jones et al. (2007) showed that supplementing the rhizobacterial community with a *Pseudomonas aeruginosa* strain influenced the fitness of the cereal aphid (*Sitobion avenae*) on barley (*Hordeum vulgare*) either positively or negatively (increased or decreased population size) depending on plant and aphid genotype. In a later study (Tétard-Jones et al., 2012) they identified genomic regions (QTLs) underlying the differential plant-mediated responses to the rhizobacterium. This linking of differential responses to genomic regions is exceptional; often studies can only indicate that factors such as genotype may be relevant for the effectiveness of PGPR-triggered plant defense responses, without further mechanistic explanation. Many studies focusing on PGPR and induced defense have been carried out under relatively sterile conditions in laboratories or greenhouses. Studies on induced defense of *P. fluorescens* under field conditions are relatively rare. In this review we do include references to biocontrol studies but want to stress that biocontrol can be the result of many mechanisms of which ISR is only one. Studies testing whether effects of particular PGPR strains on plant defense observed under sterile greenhouse conditions can be observed in the field as well have yielded mixed results (e.g., Guo et al., 2004; Akila et al., 2011). When differences are observed, these could be due to the several biotic and abiotic factors which differ between greenhouse and the field. When spatial variation in the disease suppressive effects of PGPR are observed in field trials on multiple locations, abiotic factors such as fertility, temperature, and moisture are usually discussed as explanation for the variable results (Guo et al., 2004; Ji et al., 2006; Maeder et al., 2011). Remarkably, surprisingly little attention has been paid so far to the role of biotic factors such as local plant mutualists, predators, decomposers, other pathogens, or herbivores which might interfere with the PGPR effects on plant defense.

## *Pseudomonas* COMBINED WITH OTHER *Pseudomonas* STRAINS OR WITH OTHER PLANT MUTUALISTS

Most plant species can have myriad mutualistic interactions, which provide benefits such as increasing nutrients, producing hormones, increasing tolerance to abiotic stresses (water, temperature, heavy metals), or biotic stresses (pests and pathogens). These benefits can be provided by both bacteria and fungi; depending on the mutualist species, the association can be on the root or leaf surface or inside the plants (endophytes). *P. fluorescens* is mostly known as a root colonizer. Many studies have examined the effects of adding several mutualistic organisms simultaneously to soils on plant defense (e.g., Jaderlund et al., 2008; Saravanakumar et al., 2009; Senthilraja et al., 2010). A general conclusion that can be drawn from these studies is that multiple microbial introductions typically are more effective than single introductions for biocontrol (Whipps, 2001).The combinations of species that have been added range from multiple *Pseudomonas* isolates (Saravanakumar et al., 2009; Seenivasan et al., 2012) to adding other mutualistic bacteria (Domenech et al., 2006) or mutualistic fungi (Jaderlund et al., 2008). The addition of multiple agents enhances the chance that at least one is well adapted to the local environment where the organisms are introduced. Disease suppression by the plant can also be improved when the introduced mutualists differ in their effects on induced defense responses (Domenech et al., 2006). Moreover, interactions between mutualists may lead to different gene expression and secondary metabolite production in the bacterium and this can result in synergistic effects of mutualists on the plant (Combes-Meynet et al., 2011; Garbeva et al., 2011). The studies by Garbeva et al. (2011) and Combes-Meynet et al. (2011) illustrate both the potential of bacteria species interactions to alter gene expression in *P. fluorescens*, and the potential effects that such changes in gene expression can have on the interactions with the plant. Even without altered gene expression in the bacteria, the plant may respond synergistically to the microbial-associated molecular patterns (MAMPs) of multiple plant mutualists (Desaki et al., 2012). MAMPs are molecules from pathogenic and mutualistic microbes which trigger plant immune response (van Wees et al., 2008). PGPR may vary their phenotype in order to avoid stimulation of the plants’ immune system (Zamioudis and Pieterse, 2012). It is unknown how effective such phase variation is when other PGPR are also colonizing the plant, each species with their specific MAMPs, but triggering pathways that at some point converge (van Wees et al., 2008; Zamioudis and Pieterse, 2012). Currently not enough is known about the MAMPs of *P. fluorescens* and other mutualists to speculate about synergistic effects between more different MAMPs.

### *Pseudomonas* COMBINED WITH OTHER *P. fluorescens* STRAINS

A number of studies have applied multiple strains of *P. fluorescens* to achieve better biocontrol of plant pests and pathogens, with the aim to find combinations of strains with complementary effects on plant defense. A recent study by Loper et al. (2012) on the genomes of 10 *P. fluorescens* strains shows that these strains vary considerable in their defense traits. This offers ample room for selection of complementary strains and different lifestyles. Agusti et al. (2011) selected two *P. fluorescens* strains which differed in secondary metabolite production and found that dual inoculations lead to better control of *Phytophthora cactorum* in strawberry, as well as to a reduction of the within-experiment variability, compared to single introductions. Several studies have reported that a combination of introductions of *P. fluorescens* isolates Pf1, TDK1, and PY15 is very effective in controlling pests and diseases. Introduction of the combination of the three *P. fluorescens* isolates, for example, is very effective in reducing populations of the root-feeding nematode *Meloidogyne graminicola* (Seenivasan et al., 2012) as well as in controlling sheath rot *Sarocladium oryzae* in rice (Saravanakumar et al., 2009). The explanation for the effectiveness of this particular combination is that these three isolates do not compete for space and together colonize the root surface more effectively than single isolates. This is important for the direct nematicidal effects of the isolates. However, plants inoculated with *P. fluorescens* mixtures also had higher activities of peroxidase and chitinase enzymes than single inoculations (Saravanakumar et al., 2009; Seenivasan et al., 2012), suggesting that higher activation of defense-related enzymes may play a role in addition to a more efficient occupation of the root surface.

### *Pseudomonas* COMBINED WITH OTHER SPECIES OF PGPR

The most commonly investigated combination of *P. fluorescens* and other PGPR is with *Bacillus* spp., but also combined introductions with *Burkholderia *spp., *Rhizobium* spp., and *Serratia* spp. are frequently studied. For *Bacillus* spp., as far as we are aware, no antagonistic effects on control of bacteria, fungi, and viruses have been reported. García-Gutiérrez et al.(2012) tested suppression of both fungal and bacterial pathogens by *P. fluorescens* in combination with *Bacillus*; combinations were equally effective as single introductions of *P. fluorescens.* The improved control of *Fusarium* disease by a combination of *P. fluorescens *and* Bacillus *was associated with the induction of the defense-related enzymes peroxidase and polyphenol oxidase (Akila et al., 2011; Sundaramoorthy et al., 2012). Most studies on the effects of combined introductions of *Pseudomonas *and other PGPR have reported effects on improved biological control. Combes-Meynet et al. (2011) hypothesized that during evolution PGPR have developed mechanisms to affect and respond to each other and that it is likely that the secondary metabolites from *P. fluorescens* will affect other PGPR. The authors tested the effect of 2,4-diacetylphloroglucinol (2,4-DAPG), a secondary metabolite from *P. fluorescens*, on *Azospirillum* gene expression and found that genes involved in several traits related to root colonization and growth promotion were upregulated. Co-inoculation of *P. fluorescens* and *Azospirillum* stimulated root growth in spring wheat (Combes-Meynet et al., 2011). Garbeva et al. (2011) studied changes in gene expression in *P. fluorescens* when exposed to three other rhizobacteria: *Bacillus *sp., *Brevundimonas* sp., or *Pedobacter *sp. Interestingly, *P. fluorescens *had specific responses to the different competitors; two species increased antimicrobial metabolite production by *P. fluorescens*, but *Bacillus* did not (Garbeva et al., 2011). There are also studies where inoculation with *P. fluorescens* alone was more effective than inoculations in which *Pseudomonas* was combined with other PGPR (Anwar-ul-Haq et al., 2011; Stockwell et al., 2011). *P. fluorescens* A506 proved incompatible with two other biological control agents *Pantoea vagans* and *Pantoea agglomerans *since proteases from *P. fluorescens* A506 degrade the antibiotics from the *Pantoea* spp. that play an important role in the control fire blight in pear (Stockwell et al., 2011).

### *Pseudomonas* COMBINED WITH FUNGI

Fungi are introduced together with *P. fluorescens* with three main aims: improved nutrition or plant growth (mycorrhizal fungi), improved disease control (e.g., *Trichoderma* spp.) or improved insect pest control (*Beauveria* spp.). So far, there are only a few papers that have examined the effectiveness of combined introductions of *Pseudomonas* with the entomopathogenic fungus *Beauveria.* Entomopathogenic fungi can be found as plant endophyte and may have plant growth-promoting properties (Vega et al., 2009). The majority of papers report increased control of pests or diseases when the entomopathogenic fungus *Beauveria* is applied in combination with *P. fluorescens*. Senthilraja et al. (2010) used combinations of *P. fluorescens* and *Beauveria* *bassiana* and found the three-strain combination of two *P. fluorescens* strains with one *Beauveria* *bassiana* to be more effective than single or two-strain inoculations for controlling both a leafminer and collar rot. The explanation is that *P. fluorescens* affects plant metabolism, and this, in turn, makes the insects more vulnerable to *Beauveria*. Similarly, Karthiba et al. (2010) combined *P. fluorescens* with *Beauveria* *bassiana*, and found simultaneous control of pests and pathogens on rice.

*Pseudomonas fluorescens* is known to control pathogens including fungi, and thus we may anticipate that combined effects of *P. fluorescens* and mutualistic fungi on plant resistance will be less than additive because mutualistic fungi will suffer from *P. fluorescens*. On the other hand, *Pseudomonas fluorescens* is identified as one of the mycorrhiza helper bacteria for both ecto- and arbuscular mycorrhiza (Frey-Klett et al., 2007). Mycorrhiza helper bacteria are bacteria associated with mycorrhiza that promote the symbiosis between fungus and plant by stimulating fungal growth or protecting the fungus against other fungal competitors. There are many examples where *P. fluorescens* combined with mutualistic fungi was more successful than single inoculations of either bacteria or fungi (Tayal et al., 2011; Walker et al., 2012), and antagonistic interactions have rarely been reported (but see Sukhada et al., 2011). Inoculation of the mycorrhizal fungus *Glomus intraradices *has a positive effect on *P. fluorescens* survival on maize; it is unclear whether this is a plant-mediated or direct effect (Walker et al., 2012). Sukhada et al. (2011) found both under controlled conditions and in the field that the tripartite inoculation of *P. fluorescens* with the arbuscular mycorrhizal fungi *G. mosseae* and with *T. harzianum* was not as good in reducing *Phytophthora* disease incidence as the dual inoculations. It is unknown whether the predominantly positive results of mutualistic fungi with *P. fluorescens* indicates that natural selection has favored traits that result in interactions between mutualistic fungi and *P. fluorescens* that are neutral or positive for the plant or whether this reflects the research bias towards studies using candidates with good prospects for positive interactions in their effects on biocontrol. Another major group of mutualistic fungi are the Class I endophytes *Neotyphodium* spp. and *Acremonium* spp. but little is known about their effects on belowground processes, except for a stimulation of root exudation (Omacini et al., 2012). Recently, Wicklow and Poling (2009) showed that there are negative effects of antibiotics from *Acremonium zeae* on *P. fluorescens*, but apart from that, we are not aware of any study examining the effects of plant – endophyte – *P. fluorescens* interactions on plant defense.

## INTERACTIONS BETWEEN *Pseudomonas* AND BACTERIVORES AND DECOMPOSERS

*Pseudomonas fluorescens *may also interact with two other groups of rhizosphere organisms that have less close relations with the plant: organisms which feed on bacteria (bacterivores) and organisms which break down organic material (decomposers).

### *Pseudomonas fluorescens* AND BACTERIVORES

*Pseudomonas fluorescens *are grazed by predatory bacteria, protozoa, and bacterivorous nematodes (Elsherif and Grossmann, 1996). For bacterivores there is a clear potential direct effect on *P. fluorescens* abundance via grazing. However, whether grazing will affect plant defense is dependent on the selectivity of grazing and whether induction of plant defense is density-dependent. A threshold density of *P. fluorescens* is known for effective suppression of take-all decline (Raaijmakers and Weller, 1998) but it is unclear if the same applies to other pests and diseases. Selective grazing can change bacterial competition (Pedersen et al., 2009) and bacterivores avoiding *P. fluorescens* due to the secondary metabolite production by *P. fluorescens* can improve the competitive advantage of *P. fluorescens* over other bacteria (Jousset et al., 2008; Jousset, 2012). *Pseudomonas* can produce hydrogen cyanide and this repels bacterivorous nematodes. Also 2,4-DAPG, an antibiotic compound produced by *P. fluorescens*, acts as nematicide (Neidig et al., 2011). However, even when *P. fluorescens* is grazed upon by predators, a reduction in ISR response is not self-evident, because the reduction in abundance by predation may be accompanied by other changes in the PGPR that enhance ISR. For instance, grazing by amoebae was found to upregulate 2,4-DAPG synthesis in *P. fluorescens* (Jousset and Bonkowski, 2010). This compound is also known to be directly involved in ISR in plants (Weller et al., 2012). Bacterivorous nematodes can also stimulate PGPR effects on plant growth (Jiang et al., 2012). Addition of bacterivorous nematodes together with *Burkholderia *or *Pseudomonas* to plants growing in natural soil increased microbial biomass (Jiang et al., 2012), indicating a stimulation of bacterial abundance by grazing. Both nematode addition and *Burkholderia *addition increased the number of root tips, but their combined effect was significantly higher than their single effects (Jiang et al., 2012). There is an urgent need for experiments, that include bacterivores, *P. fluorescens*, plants, and pathogens in which the expression of plant defense or defense genes are measured. We are not aware of any of such studies.

### *Pseudomonas fluorescens* AND DECOMPOSERS

For decomposers more and more evidence is accumulating that they can affect induced defense responses of plants. For instance, Collembola induce auxin-responsive genes and defense genes in shoots of *Arabidopsis* (Endlweber et al., 2011), although in this case Collembola may act as herbivores instead of decomposers. Earthworms reduced the damage by plant parasitic nematodes in rice, without directly affecting nematode abundance (Blouin et al., 2005). The exact mechanism is unknown, but earthworms did modulate expression of three stress-related genes and they also improved the photosynthetic capacity of the plant (Blouin et al., 2005). The presence of earthworms in the soil can also cause an increase or decrease in defensive glucosinolates in Brassicaceous plants (Wurst et al., 2006; Lohmann et al., 2009; González Megías and Müller, 2010). These results clearly show that decomposers can affect plant defense and therefore, decomposers may interact with *P. fluorescens*-mediated ISR. Indirect effects of decomposers on *P. fluorescens*–plant outcomes may occur via changes in nutrient availability and substrate quality, and several studies have indicated that soil nutritional conditions are crucial for ISR (e.g., Hoitink and Boehm, 1999). It is also possible that decomposers affect plant growth and root exudation, and that this in turn affects *P. fluorescens* abundance and ultimately plant defense. However, we are not aware of any study describing effects of decomposers on *P. fluorescens*–plant interactions. For other PGPR it has been shown that earthworm casts increased PGPR abundances (Wu et al., 2012). Jana et al. (2010) investigated the effects of earthworms on *Arabidopsis thaliana* in nutrient poor and rich soil. Since earthworms affected several plant parameters independent of soil nutrient conditions, the authors suggested that earthworms stimulate nutrient mineralization but also stimulate phytohormone-producing bacteria (Jana et al., 2010). In another study, earthworms increased the abundance of fluorescent pseudomonads in the rhizosphere of three plant species (Elmer, 2009), and therefore these results suggest that this may be a general phenomenon. The mechanism of stimulation is unknown, but Troxler et al. (2012) observed that earthworms provide survival hotspots for *P. fluorescens* in soil.

## SYNTHESIS, APPLICATION, AND OUTLOOK

**FIGURE 1:**
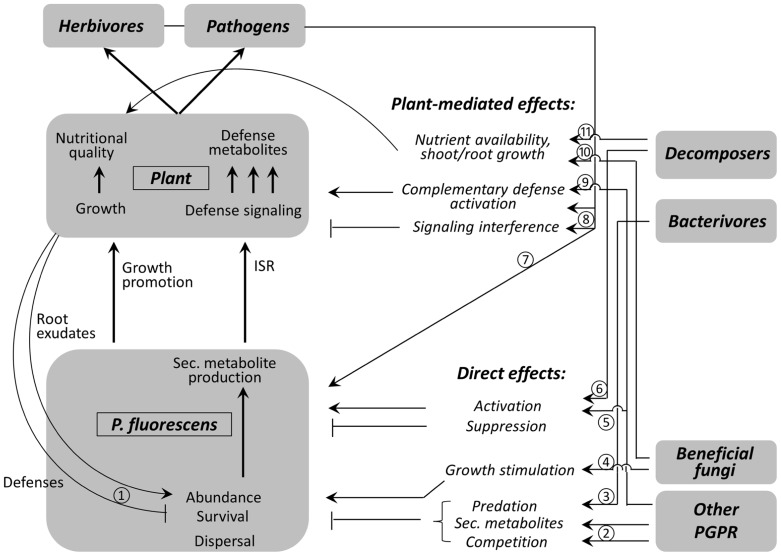
**Simplified scheme of direct and indirect (plant-mediated) effects of rhizosphere- and plant-associated organisms on interactions between *Pseudomonas fluorescens* and host plant defenses.** Arrows: enhancing (positive) effects, lines ending with small vertical bars: suppressive (negative) effects. For simplicity, reciprocal effects (effects of *P. fluorescens* on other organisms) have not been included. Circled numbers refer to articles describing the interactions: (1) Jin et al. (2010), (2) Gu (2009); Prieto et al. (2011), (3) Elsherif and Grossmann (1996); Pedersen et al. (2009), (4) Walker et al. (2012), (5) Combes-Meynet et al. (2011); Garbeva et al. (2011), (6) Elmer (2009); Troxler et al. (2012), (7) Jousset et al. (2011), (8) Dicke et al. (2009); Zhang et al. (2013), (9) Elbadry et al. (2006), (10) Jones et al. (2004), and (11) Wurst (2010).

The effect of other soil dwelling organisms on the impact of *P. fluorescence* on plant defense responses will depend on whether there is a threshold density and whether the effects are (linear or non-linear) density-dependent. If other PGPR organisms target the same ISR mechanism then one could easily imagine additive interactions if there is a linear relationship between density at introduction and the effect on the plant. If the relation between density and plant response is non-linear there is room for synergistic reactions. There are several mechanisms by which the presence of other organisms can influence interactions between *P. fluorescens, *host plants, and herbivores or pathogens (**Figure 1**). The other species can act directly via affecting abundance or effectiveness of *P. fluorescens *and indirectly via plant-mediated effects. The direct effects can be separated into quantitative and qualitative effects: the quantitative effects are those that determine the number of *P. fluorescens* cells in the rhizosphere. The qualitative effects determine the effectiveness of *P. fluorescens *in triggering plant defense, e.g., by changing 2,4-DAPG production. The number of *P. fluorescens* cells can be decreased due to predation by bacterivores, such as predatory bacteria, nematodes, and protozoa (**Figure 1**; Pedersen et al., 2009). *Pseudomonas fluorescens* is a suitable food source for bacterivores (Elsherif and Grossmann, 1996), but *P. fluorescens* can produce defense compounds to avoid predation. The overall effect of grazing on *P. fluorescens* population dynamics will depend on the defense levels of *P. fluorescens*, the availability of alternative food sources and the selectivity of the grazers. We do not foresee an immediate application of combining *P. fluorescens* inoculation with bacterivores to increase root colonization.

Competition for nutrients or space with other PGPR or rhizobacteria is another factor that will negatively affect *P. fluorescens* numbers (**Figure 1**; Prieto et al., 2011). Other PGPR could also produce secondary metabolites which inhibit *P. fluorescens *(**Figure 1**; Gu, 2009). Many efforts have been made to isolate and screen *P. fluorescens* strains and to select successful combinations of multiple *P. fluorescens* strains or other PGPR. Recent developments have now enabled screenings of *P. fluorescens* genomes for defense traits and this could help with selection of compatible and potential synergistic strains. Synergistic interactions between *P. fluorescens* and other plant mutualists are much more common than antagonistic interactions, but this may be due to a bias in experimental studies to use species with prospects of positive interactive effects on biocontrol. Only the most promising strains are selected and these are first tested for compatibility, i.e., absence of *in vitro* inhibition (e.g., Sundaramoorthy et al., 2012) or competition during root colonization (Prieto et al., 2011). This approach probably provides a biased view of the effects of interactions between mutualist species on plant defense. For inoculation approaches this screening approach is very efficient, but for understanding interactions between introduced *P. fluorescens* with the resident biocontrol agents knowledge of a broader range of species interactions is necessary. Application of single *P. fluorescens* requires knowledge on potential interactions with resident *P. fluorescens* and other organisms. At least 22 genotypes of 2,4-DAPG-producing *P. fluorescens* have been detected thus far; multiple isolates are found together in soils (Weller et al., 2007) and thus interactions between resident and inoculated *P. fluorescens* are likely to occur. Also other PGPR are widespread and they can interact with introduced *P. fluorescens*. Those groups that negatively affect *P. fluorescens *abundance (bacterivores and other PGPR) may also trigger secondary metabolite production in *P. fluorescens *(qualitative effect). Those secondary metabolites such as 2,4-DAPG serve as defense compounds against predators but are also involved in ISR in plants (Weller et al., 2012). Upregulation of such inducing compounds probably lowers the threshold density necessary to induce ISR in plants. Garbeva et al. (2011) showed how some PGPR increased secondary metabolites production in *P. fluorescens* while other PGPR did not change secondary metabolite production. This variation in interactions allows for selection of compatible PGPR combinations, but prediction of field effects due to interactions with resident species will remain problematic. For most field situations there is no knowledge of the resident *P. fluorescens* and other PGPR. The fast developments in molecular techniques continuously improve the resolution at which microbial community composition can be assessed. Even when composition of the local bacterial community is known, this knowledge would be of little use when for most species nothing is known about their potential interaction with introduced *P. fluorescens.* Metagenomics and transcriptomics will offer insight in activity and function of the microbiome, especially the recruitment and activation of beneficials (Berendsen et al., 2012).

Both decomposers (Elmer, 2009; Troxler et al., 2012) and mycorrhizal fungi (Walker et al., 2012) can have positive effects on the number of *P. fluorescens* cells in the rhizosphere. However, there is only a single report for stimulation of *P. fluorescens* survival by mycorrhizal fungi and hence the generality of this phenomenon remains unclear. The exact mechanism of *P. fluorescens* stimulation by decomposers and mycorrhiza is still unknown. However, regardless of the mechanism, the positive effect of decomposers and possibly mycorrhizal fungi on *P. fluorescens *abundance and dispersal could be exploited by adapting management practices to, e.g., stimulate earthworms by organic amendments. Tillage and fertilization could be adapted to favor arbuscular mycorrhizal fungi. Apart from promoting PGPR, it is clear that there are many other reasons why decomposers or arbuscular mycorrhizal fungi are stimulated in agricultural soils, although such management practises are often not yet adopted in intensive agricultural systems.

Indirect effects via plant feedback comprise a multitude of interactions. Decomposers and beneficial fungi may increase nutrient availability and increase shoot and root growth (Laakso and Setälä 1999; Jones et al., 2004). The increased growth may change plant defense and root exudation patterns and this, in turn, can affect *P. fluorescens *populations (Jin et al., 2010). With the recently increased awareness of the indirect effects of decomposers on plant defense (e.g., Wurst, 2010), the interaction between *P. fluorescens, *plants, and plant–decomposer is a research area waiting to be explored.

Although there certainly is interest in the control of multiple pests or pathogens simultaneously (Karthiba et al., 2010; Senthilraja et al., 2010), most experiments thus far have tested the effect of *P. fluorescens* on control of pathogens separately. Experiments, where the effect of one pathogen on the interaction with another pathogen and *P. fluorescens* has been studied, are scarce. Pathogens, mutualists, and pests with intimate relationships with the plant such as aphids or cyst nematodes might be able to synergize or antagonize the ISR-triggered responses through interference with defense signaling or activation/repression of downstream defenses. The pathogen *Pythium ultimum* can change 2,4-DAPG production by *P. fluorescens *(Jousset et al., 2011), but it is currently unknown how that would affect a second attacker. For insects, interspecific asymmetrical competition is frequently found (Kaplan and Denno, 2007). Therefore, control of one insect pest could result in an increase in abundance of another. Some plant–nematode combinations are sensitive to *P. fluorescens*, but not all (Timper et al., 2009). Thus, similar to insects, controlling one nematode pest might affect the abundance of other nematode species for example by changing competition for root space (Brinkman et al., 2004). A single plant which is attacked by multiple herbivores, pathogens or herbivores and pathogens would have to deal with a number of possible conflicting signals. A second attacker could activate or repress downstream defenses induced by a previous attacker (Dicke et al., 2009; Zhang et al., 2013). In this respect the order by which a plant is attacked is crucial for plant defense induction. Priority effects are receiving increasingly more attention recently, also in the context of plant–soil interactions, but mostly from the plant perspective. However, there is very little empirical work on the interactions between *P. fluorescens*, a host plant, and multiple attackers. In fact, empirical studies that examine how interactions between herbivores, pathogens, mutualists, decomposers, or bacterivores affect plant–*P. fluorescens* interactions in a full-factorial design are non-existing and can only be addressed by individual-based models of plant-based multitrophic species interactions, such as in Meyer et al. (2009). We conclude that other rhizosphere inhabitants can greatly influence *P. fluorescens* and its interactions with the plant. However, there is still a dearth of information about the effects of other species on interactions between *P. fluorescens *and plant defense. Insight into these interactions will contribute to improved performance of biocontrol agents in the field.

## Conflict of Interest Statement

The authors declare that the research was conducted in the absence of any commercial or financial relationships that could be construed as a potential conflict of interest.
